# Supplementary material for: Secreted HSP90α-LRP1 Signaling Promotes Tumor Metastasis and Chemoresistance in Pancreatic Cancer
Source: Int J Mol Sci. 2022 May 16;23(10):5532. doi: 10.3390/ijms23105532 (PMC9141888; doi:10.3390/ijms23105532)
Supplement: Supplementary file 1 [file ijms-23-05532-s001.zip › ijms-1701289-supplementary.pdf]

Secreted HSP90α-LRP1 signaling promote tumor metastasis and chemoresistance in pancreatic cancer

Nina Xue[1], Tingting Du[1], Fangfang Lai, Jing Jin, Ming Ji*, Xiaoguang Chen*

State Key Laboratory of Bioactive Substances and Functions of Natural Medicines & Beijing Key Laboratory of Non-Clinical Drug Metabolism and PK/PD study, Institute of Materia Medica, Chinese Academy of Medical Sciences and Peking Union Medical College, Beijing 100050, China

[1]These authors contributed equally to this work.

*Correspondence to: Ming Ji and Xiaoguang Chen

E-mail address: Jiming@imm.ac.cn (M. Ji); chxg@imm.ac.cn (X. Chen)

Acknowledgements

This project was supported by the National Natural Science Foundation of China (81573466).

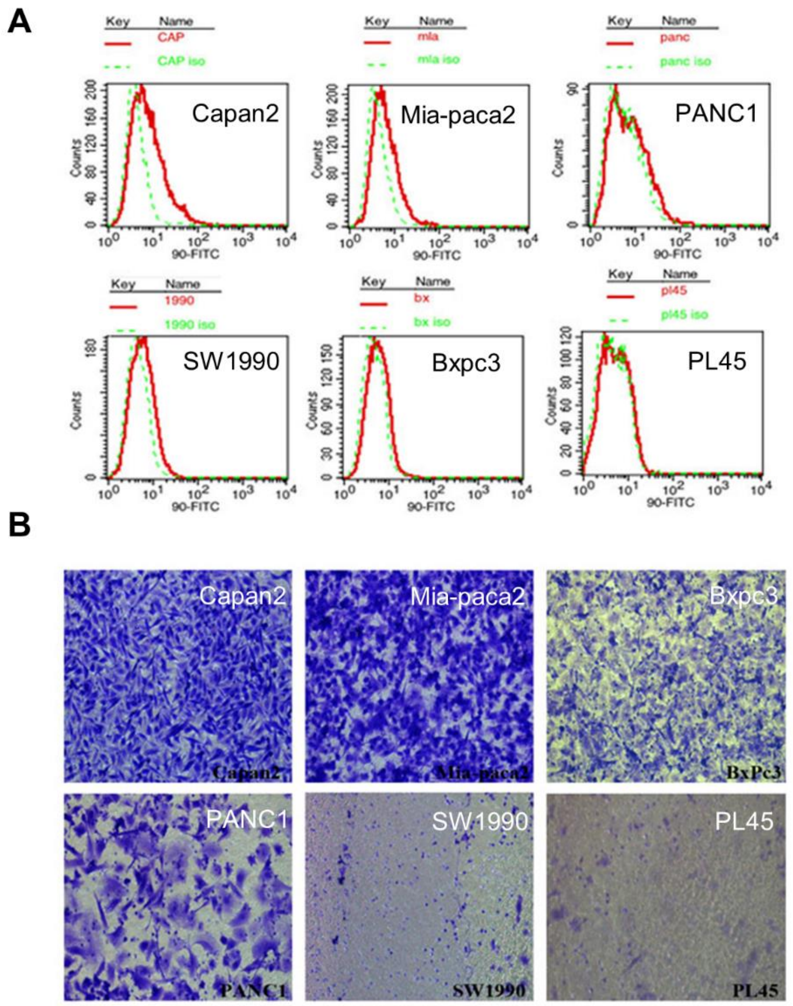

Figure S1. Membrane HSP90α expression and invasive ability of PC cells. **(A)** The cell surface HSP90α levels of Capan2, Mia-paca2, PANC1, SW1990, Bxpc3 and PL45 cells after serum starvation were detected using flow cytometry analysis. **(B)** The invasive abilities of above-mentioned PC cells were detected by transwell assay.

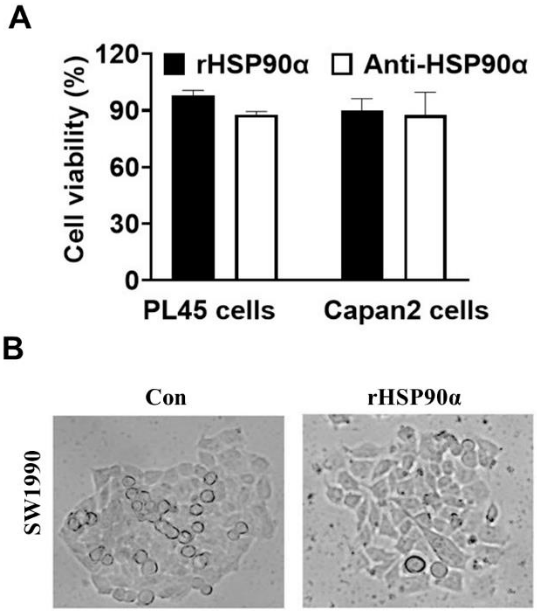

Figure S2. Cell viability and cellular morphology of PC cells. **(A)** The cell viability of PL45 and Capan2 was determined by MTT assay after exposure of (10 μg/mL) rHSP90α or (5 μg/mL) anti-HSP90α antibody for 72 h. **(B)** Images of SW1990 cells morphology were measured under a microscope after stimulated by (10 μg/mL) rHSP90α for 72 h.

methods

Flow cytometry assay

The human PC cell lines including Capan2, Mia-paca2, SW1990, Bxpc3, PANC1 and PL45 were harvested and resuspend at a density of approximately 2×10^6 cells/ mL in ice cold PBS. The cells were stained with 10μg/mL of Mouse monoclonal to HSP90α antibody (ab79849) in 3% BSA/PBS and incubated for 1 h at 4°C. After washing for three times, the cells were incubated in the appropriate Alexa Fluor® 488 secondary antibody (1:500 dilution) for 30 minutes on ice in the dark. After washing, the membrane-bound HSP90α expressions were measured by Beckman coulter flow cytometry. The samples stained with only fluorochrome-labeled secondary antibody were as negative control.
